# Dosimetry in ^177^Lu-PRRT for Neuroendocrine Tumors: Current Concepts, Clinical Relevance and Future Perspectives

**DOI:** 10.3390/jcm15134952

**Published:** 2026-06-25

**Authors:** Małgorzata Elżbieta Poniatowska-Roszkowska, Tabea Troschke, Bożena Birkenfeld, Hanna Piwowarska-Bilska

**Affiliations:** 1 Department of Nuclear Medicine, Pomeranian Medical University in Szczecin, 70-204 Szczecin, Polandhanna.piwowarska.bilska@pum.edu.pl (H.P.-B.); 2 Department of Pediatrics, Division of Pediatric Hematology and Oncology, Greifswald University Hospital, 17475 Greifswald, Germany

**Keywords:** dosimetry, NETs, lutetium-177, systemic radionuclide therapy

## Abstract

**Background:** Neuroendocrine tumors—are relatively rare but increasingly diagnosed malignancies originating from diffuse neuroendocrine cells, most commonly affecting the gastroenteropancreatic system. Due to their long asymptomatic development and low incidence, pose a diagnostic and therapeutic challenge for physicians. Recently, the role of nuclear medicine has been growing not only in the diagnostic stage but also in treatment. Systemic radionuclide therapy using somatostatin analogs labelled with the radioisotope lutetium-177 is becoming increasingly common in patients with advanced-stage disease. Currently, most patients receive a standard activity of therapeutic radiopharmaceuticals. Recent clinical studies provide increasing evidence of a close relationship between the absorbed radiation dose in pathological lesions and the therapeutic effect of radioisotope therapy. Internal dosimetry is used to measure the doses of ionising radiation absorbed by the patient after administration of the radiopharmaceutical. The lack of individual internal dosimetry prior to therapy means that only a small fraction of patients receive optimal doses of radioactivity, which is markedly different from external beam radiotherapy planning. **Methods:** A narrative literature review was conducted using the PubMed/MEDLINE and Embase databases, focusing primarily on publications from the last years. The search strategy included combinations of keywords related to peptide receptor radionuclide therapy and dosimetry, such as “Lutetium-177”, “neuroendocrine tumors”, “dosimetry”, “PRRT”, “systemic radionuclide therapy” and “artificial intelligence”. Particular emphasis was placed on recent prospective clinical studies, multicenter investigations, systematic reviews and consensus documents published by major nuclear medicine societies, including the European Association of Nuclear Medicine (EANM) and the Society of Nuclear Medicine and Molecular Imaging (SNMMI). Seminal earlier publications considered essential for understanding the development of dosimetry concepts and clinical implementation were also included. **Results:** This study confirms the existence of a clinically significant dose-response relationship in ^177^Lu-PRRT. Higher absorbed doses to tumour lesions are associated with longer progression-free survival. The lack of individualized internal dosimetry prior to therapy means that only a small proportion of patients receive optimal radiation doses. Simplified dosimetric approaches with a reduced number of imaging time points, together with emerging artificial intelligence–based tools, appear promising for reducing the complexity of the dosimetry process. **Conclusions:** The aim of this study was to analyse the current literature on the role of internal dosimetry in the treatment of neuroendocrine tumors using the radioisotope lutetium-177. Available data support the clinical relevance of individualized dosimetry and highlight its potential to optimize both therapeutic efficacy and treatment safety.

## 1. Introduction

Neuroendocrine tumors (NETs) are neoplasms originating from diffuse neuroendocrine cells. In approximately 70% of cases, they develop within the gastrointestinal tract and pancreas. Although the overall incidence is relatively low, it has been steadily increasing and is currently estimated at approximately 7 per 100,000 individuals [1].

All NETs are malignant, but they vary in aggressiveness depending on the degree of cellular differentiation and histological maturity. These parameters are assessed by the Ki-67 proliferation index and/or mitotic count, according to WHO classification. The 2022 WHO classification categorizes well-differentiated neuroendocrine tumors into three grades based on the Ki-67 proliferation index: G1 (Ki-67 < 3%), G2 (Ki-67 3–20%) and G3 (Ki-67 > 20%). In addition, poorly differentiated neuroendocrine carcinomas (NECs) are recognized as a separate entity. Tumour grade remains the most important prognostic factor and plays a central role in treatment selection, including qualification for peptide receptor radionuclide therapy (PRRT) [2].

Neuroendocrine tumors may be hormonally active or non-secreting. In the clinical presentation of gastrointestinal NETs, symptoms are often related to hormone or amine overproduction. Due to their often indolent and asymptomatic growth, NETs are frequently diagnosed at an advanced stage of the disease [3].

Diagnostic imaging modalities as ultrasonography, computed tomography (CT) and magnetic resonance imaging (MRI) play a key role in the diagnostic process [4,5].

Radionuclide imaging is considered the most sensitive diagnostic tool for patients with tumors expressing somatostatin receptors. It is used to identify the primary tumor site, stage the disease, qualify patients for treatment with somatostatin receptor (SSTR) analogs and to monitor treatment response. Visualization of somatostatin receptor expression is typically performed using SPECT/CT or PET/CT imaging with radiolabeled SST analogs [6]. PET/CT using ^68^Ga-labelled somatostatin analogues (DOTATATE, DOTATOC and DOTANOC) and SPECT/CT with technetium-99m Tektrotyd are the standard functional imaging modalities for neuroendocrine tumors. These techniques enable sensitive assessment of somatostatin receptor expression, which is essential for patient selection for PRRT. Eligibility for treatment requires radiotracer uptake in all target lesions that is at least equal to physiological liver uptake [7,8].

Therapeutic strategies depend on tumor grade, differentiation and disease stage. Well-differentiated NETs with a low Ki-67 proliferation index and confirmed somatostatin receptor expression may qualify for treatment with somatostatin analogs (SSA) [9].

Patients with advanced, unresectable or metastatic SSTR-positive disease may be eligible for PRRT. This treatment involves the intravenous administration of radioactive somatostatin analogs, labeled with the radionuclides yttrium-90 (^90^Y) or lutetium-177 (^177^Lu), delivering targeted ionizing radiation to tumor cells [10].

Physical and biological basis of therapy and dosimetry in ^177^Lu-based PRRT.

The therapeutic agents used in PRRT are somatostatin analogs labeled with a beta emitter, either yttrium-90 (^90^Y) or lutetium-177 (^177^Lu). These two radionuclides differ substantially in their physical properties, with important consequences for clinical use and dosimetric assessment. ^90^Y is a pure beta-emitter (Emax 2.27 MeV, mean tissue range ~2.5 mm, maximum ~11 mm), which may be advantageous for treatment of larger, bulky tumors. However, the absence of significant gamma emission makes post-therapy imaging-based dosimetry technically challenging, typically requiring bremsstrahlung SPECT or ^90^Y PET/CT based on the low-abundance beta-plus emission [11]. In contrast, ^177^Lu emits lower-energy β-particles (E_max 0.497 MeV; mean tissue range ~0.67 mm) together with gamma photons at 113 and 208 keV, enabling high-quality quantitative SPECT/CT imaging after each treatment cycle and facilitating patient-specific dosimetric assessment [12].

Lutathera^®^, a radiopharmaceutical labeled with ^177^Lu, was approved by the European Medicines Agency (EMA) in 2017 and by the U.S. Food and Drug Administration (FDA) in 2018 as the first agent for PRRT. The treatment regimen typically consists of four intravenous 7.4 GBq infusions of the radiopharmaceutical administered at 6–8 week intervals, performed on an outpatient, single-day basis. To protect renal function, a slow intravenous infusion of an amino acid solution containing L-lysine and L-arginine should be initiated 30 min prior to each Lutathera^®^ administration. Dose modifications may be required based on laboratory parameters and clinical status [12,13].

The NETTER-1 trial demonstrated that ^177^Lu-based PRRT significantly improves progression-free survival and quality of life compared with high-dose octreotide LAR in patients with well-differentiated gastrointestinal neuroendocrine tumors, confirming its safety and clinical efficacy in advanced disease [14].

Despite these favorable outcomes, there is substantial interindividual variability in radiopharmaceutical biodistribution. Consequently, further optimization of dosing protocols and a comprehensive evaluation of therapeutic efficacy in relation to treatment-related toxicity are required, highlighting the growing importance of dosimetry-informed PRRT [15].

In many centres internal dosimetry in ^177^Lu-PRRT is based on quantitative SPECT/CT imaging performed at three time points following administration of the radiopharmaceutical—at 24, 96 and 192 h. Accurate image quantification requires prior calibration of the system using phantom measurements, as well as particular attention to reconstruction parameters, including attenuation and scatter correction. Segmentation of tumour lesions and critical organs—the kidneys, spleen and bone marrow—can be performed manually, semi-automatically or using automated tools. The choice of segmentation method is one of the main sources of variability in absorbed dose estimates [16,17].

In clinical practice, three main dosimetric approaches are currently used. MIRD (Medical Internal Radiation Dose) calculates average absorbed doses at the organ level using S-values and time-integrated activity, offering simplicity and computational efficiency at the expense of spatial resolution. Voxel-based dosimetry extends this approach to the level of the lesion by generating three-dimensional dose maps, thereby capturing dose heterogeneity within the tumour. Monte Carlo simulation provides the highest accuracy by modelling the transport of individual particles, but requires significant computational resources and remains largely confined to research environments [11,18]. The choice of dosimetric method, segmentation approach and strategy for fitting the time-activity curve influences estimates of absorbed dose; standardisation of these parameters across all centres is a prerequisite for the successful implementation of personalised dosimetry in clinical practice [16,17,18,19].

**Figure 1:**
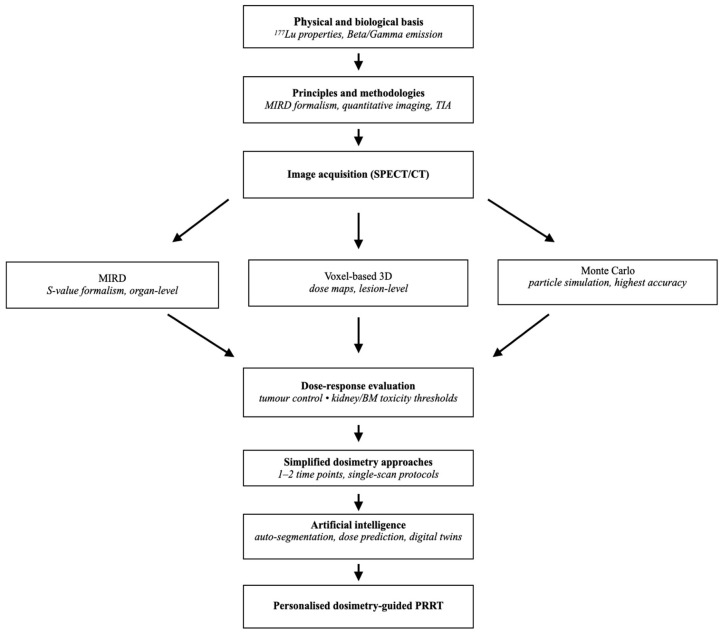
Schematic workflow of personalized dosimetry in ^177^Lu-DOTATATE PRRT. After administration of the radiopharmaceutical, serial quantitative SPECT/CT imaging is performed at multiple time points to characterize radiotracer kinetics. Segmentation of tumour lesions and organs at risk allows generation of time–activity curves and absorbed dose calculations using MIRD-based, voxel-based, or Monte Carlo approaches. Dosimetric results can subsequently be used to optimize treatment planning and support personalized radionuclide therapy.

The general workflow of personalized dosimetry in ^177^Lu-PRRT, from radiopharmaceutical administration and image acquisition to absorbed dose calculation and clinical interpretation, is illustrated in Figure 1.

## 2. Simplified Dosimetry Approaches and Artificial Intelligence in ^177^Lu-PRRT

### 2.1. Simplified Dosimetry Methods

Despite its recognised clinical value, dosimetry in PRRT using ^177^Lu is not routinely implemented in centres treating patients. This is mainly due to the complexity and time-consuming nature of standard protocols, which are difficult to implement in everyday clinical practice. Consequently, recent efforts have focused on developing simplified dosimetric methods and introducing solutions based on artificial intelligence.

Currently, in most nuclear medicine departments worldwide, fixed-activity regimens of therapeutic radiopharmaceuticals are administered to patients [20]. However, numerous clinical studies provide increasing evidence of a strong correlation between the absorbed dose of the radiopharmaceutical in pathological lesions and the therapeutic outcome of systemic radionuclide therapy [21,22,23].

Internal dosimetry refers to the measurement of ionising radiation doses absorbed by the patient following radiopharmaceutical administration. It allows for the assessment of radiation exposure to specific organs and tissues during radionuclide therapy [24]. In the absence of individualised internal dosimetry based on quantitative analysis of serial diagnostic images, only a small fraction of patients receive the optimal radiopharmaceutical activity. The vast majority receive radiation doses that are too low. While this cautious approach helps ensure the radiation safety of healthy tissues, it also results in lower doses delivered to tumour tissue, potentially leading to reduced therapeutic response and higher recurrence rates [25]. Radionuclide therapies not preceded by patient-specific dosimetric assessment are considered of a lower standard compared to external beam radiotherapy.

Personalised planning of radionuclide therapy has great potential to become a new global standard of care. The development of such individualised approaches depends heavily on the generation of robust scientific evidence supporting the superiority of personalised radionuclide therapy over fixed-activity regimens. The cornerstone of this approach is internal dosimetry, which involves calculating absorbed radiation doses in individual tissues. These dose estimations are based on the analysis of serial scintigraphic images, which are routinely acquired during the diagnostic and therapeutic process in nuclear medicine departments.

**Figure 2:**
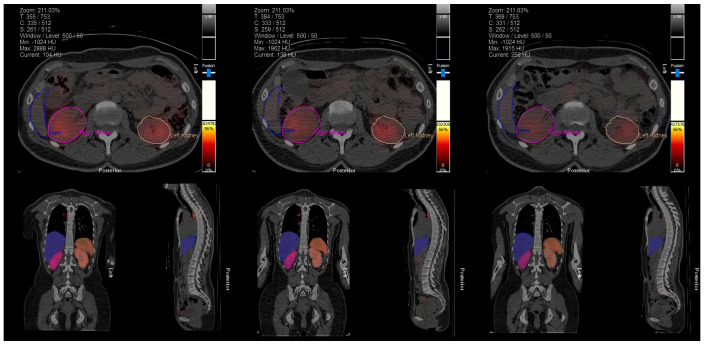
Example of image-based dosimetry in a patient undergoing ^177^Lu-DOTATATE PRRT. Quantitative SPECT/CT acquisitions were performed at 24, 96 and 192 h after radiopharmaceutical administration. The liver was segmented in blue, the left kidney in yellow, the right kidney in pink and the spleen in orange.

Currently, clinical internal dosimetry is practiced primarily in nuclear medicine centres that combine clinical care with scientific research. The implementation of patient-specific internal dosimetry for radionuclide therapy planning remains one of the main challenges in contemporary nuclear medicine [26,27,28]. Representative examples of serial SPECT/CT acquisitions and image-based dosimetric analysis are presented in Figure 2.

It is worth noting that individual patient dosimetry in nuclear medicine is more difficult to perform than dosimetry in external beam radiotherapy. In external radiotherapy, the dose is planned and controlled before the procedure and the beam geometry and energy are known. In internal dosimetry, it is necessary to reconstruct the dynamic, patient-specific distribution of the radionuclide over time and space and to account for radioactive decay and biological processes. This requires complex imaging and modelling and is associated with greater uncertainty. Dosimetric calculations based on serial scintigraphic imaging in radionuclide therapy are complex procedures [20]. Acquiring a full set of quantitative scintigraphic images requires patient availability at multiple time points, additional scanner usage (SPECT/CT or PET/CT) and extended acquisition time for nuclear medicine technologists. The acquired image series must then be analysed by trained physicists or nuclear medicine specialists using dedicated dosimetry software.

In recent years, an increasing number of studies have highlighted the clinical relevance of dosimetry in PRRT. The dose–response relationship deserves particular attention-higher absorbed doses may be associated with better tumour control. At the same time, other studies emphasise the importance of individually tailoring radiation exposure based on the dose delivered to critical organs, such as the kidneys and bone marrow, pointing to the potential of dosimetry in balancing treatment efficacy and the risk of toxicity. Despite these promising results, the implementation of dosimetry in routine clinical practice remains inconsistent [29,30,31,32,33]. Warfvinge et al. suggested that an accumulated tumour absorbed dose of ≥135 Gy was associated with an approximately 90% probability of partial response in G2 NETs, providing one of the first proposed quantitative dose thresholds relevant to treatment planning [31]. Maccauro et al., in the LUTADOSE trial, showed that tumour dosimetry after the first treatment cycle was associated with progression-free survival in patients with G1–G2 GEP-NETs treated with ^177^Lu-DOTATATE [32]. These findings indicate that early dosimetry may help identify patients who are more likely to benefit from treatment and support the concept of dosimetry-guided treatment adaptation. However, proposed dose thresholds vary between studies and patient populations and prospective validation in larger multicentre cohorts remains necessary before dose-based treatment individualisation can be recommended as standard practice. Taken together, these studies illustrate the growing clinical relevance of internal dosimetry in PRRT and its emerging role in individualised treatment planning, as summarised in Table 1.

Specialised dose calculation tools (software for internal dosimetry) can be divided into commercial and academic programs. Commercial internal dosimetry software is intended for clinical use and comes with certification, but it is usually expensive and may not account for measurement uncertainties in its calculations. In contrast, academic software is typically free, open-source and well tested; however, it is often insufficiently documented, heavily reliant on individual developers and characterised by a poor graphical user interface, making it unsuitable for routine clinical use.

Commercial dosimetry software constitutes a significant financial investment for departments and hospitals. Employing trained medical physicists or nuclear medicine professionals to perform internal dosimetry poses an additional logistical and economic challenge. If the gamma camera is not integrated with dosimetry software, external tools must be calibrated manually, adding to the complexity. Moreover, there is a lack of standardised protocols for image quantification and SPECT/CT system calibration for quantitative acquisitions.

Currently, several commercial software platforms for internal dosimetry are available on the medical market [30,31]. These tools must be integrated with SPECT/CT or PET/CT scanners. Quantitative image analysis using such software requires prior acquisition of phantom data and computational calibration procedures to ensure compatibility between imaging systems and external analysis tools. Performing this full set of physics tests and analytical calculations is essential for obtaining reliable quantitative dosimetry results in radionuclide therapy [34,35,36].

With the development of diagnostic methods and the earlier implementation of radioisotope therapy using lutetium-177, there has been an increase in the number of publications on dosimetry methods and the safety of PRRT. A 2022 study by Harris et al. confirmed the long-term safety of PRRT in patients with NETs. The organs most vulnerable to adverse effects are the kidneys and bone marrow. The most common complication is transient thrombocytopenia, which usually occurs 4–6 weeks after a treatment cycle and resolves before the next dose. Cases of long-term myelodysplasia or leukaemia were observed in slightly more than 2% of patients [37].

In the phase II LUMEN clinical trial evaluating the efficacy of ^177^Lu-DOTATATE therapy in patients with advanced, well-differentiated gastroenteropancreatic neuroendocrine tumors (GEP-NETs), dosimetry was performed after each treatment cycle for the kidneys, bone marrow and spleen. The most frequently reported adverse events were anaemia and lymphopenia (65%), thrombocytopenia and fatigue (51%), alopecia (46%) and nausea (41%). No correlation was observed between progression-free survival and decline in renal function or haematologic toxicity during PRRT. Importantly, no association was found between the absorbed dose in the bone marrow and spleen and the severity of haematologic toxicity [38].

One of the key challenges in modern nuclear oncology is accurate imaging of NET patients using somatostatin receptor (SSTR) markers prior to PRRT, as well as the administration of targeted treatment with individually adjusted radiation doses [39]. This personalised approach enhances therapeutic effectiveness and reduces the risk of side effects.

Unfortunately, currently available dosimetry methods are complex and time consuming. They require a series of imaging studies at different time points during therapy and advanced computational analysis, which limits their use to selected centres and often to a restricted extent. In addition, patients are not always able to attend subsequent imaging examinations and the process requires significant equipment resources (e.g., gamma camera time) and substantial involvement of the medical team.

The process of calculating the dose absorbed by critical organs and its changes during therapy is time consuming. In the first stage, it is necessary to delineate regions of interest (ROIs), which can be performed separately for each imaging study or once on an image from a single time point and then transferred to the remaining scans [40]. The second approach saves considerable time; however, differences in patient positioning and respiratory motion may affect the accuracy of the estimated absorbed dose.

Studies comparing planar imaging-based dosimetry with hybrid imaging-based dosimetry have shown that the latter method is more accurate in assessing doses absorbed by critical organs and tumors. The main reason for these differences was identified as the overlap of anatomical structures, with the greatest discrepancies observed in spleen dosimetry, reaching up to 52% [41]. Similar conclusions were reported by Bronsch-Lenz J. et al. and Rosar F. et al., who indicated that dosimetry based on SPECT/CT images is a more accurate method for assessing dose distribution [40,41]. In addition, the authors emphasise the need to standardise the segmentation process and to select appropriate methods that reduce variability in absorbed dose estimates for organs and lesions [16,17].

Veregnaud L. et al. indicate possible simplifications that reduce the number of acquisitions and shorten imaging time while maintaining acceptable accuracy [42]. They propose, among other approaches, limiting the number of time points to two, with acquisitions at 24 and 168 h after administration of ^177^Lu-DOTATATE. When two acquisitions are not feasible, a single examination performed between 72 and 96 h after administration of the radiopharmaceutical is recommended [42,43,44]. In addition, the authors [40] recommend the use of 360° CZT gamma cameras to reduce acquisition time; however, this may be difficult to implement due to financial constraints.

The latest paper published as part of the SNMMI Dosimetry Challenge 2024 compared dosimetry results depending on the method used (organ-level dosimetry using S values, 3D voxel dosimetry, local deposition and Monte Carlo simulations) and the type of software (commercial or open-source). Only slight differences were observed, reaching a maximum of 7%. The authors identified the method of image segmentation and the selection and fitting of activity–time curves as the key factors influencing the results [18].

### 2.2. Artificial Intelligence in Dosimetry

The use of artificial intelligence (AI) offers an opportunity to simplify and automate dosimetry processes, enabling their routine implementation in clinical practice [45]. The development of deep learning (DL) provides new opportunities for solving complex problems, including predicting dose distribution before therapy initiation and personalising radiopharmaceutical dosing. The integration of AI with the concept of digital twins creates the prospect of fully personalised treatment, which may translate into improved clinical outcomes [46].

In a 2022 study, the authors presented automatic segmentation of whole-body images from 18F-FDG PET/CT using MOOSE (Multiple-Organ Objective Segmentation) software version 2.0. The segmentation was based on the nnU-Net architecture, one of the most advanced neural networks in medical imaging. Comparison with manual segmentation showed very high agreement—for tissues outside the central nervous system (CNS), a Dice coefficient of up to 92% was achieved. This approach aligns with the development of personalised nuclear medicine and may significantly improve planning of radioisotope therapies [47].

The use of deep learning models, including U-Net, GANs and transformer architectures, significantly improves the accuracy and efficiency of dose calculations compared to traditional organ-based and voxel-based methods. Their applications in organ segmentation, image synthesis and dose distribution prediction are particularly important, enabling safer and more effective therapy planning. However, there is still a lack of clinical data and standardisation and validation of AI models are necessary to enable their reliable use in clinical dosimetry [48].

Most published models have been developed and validated using single-centre datasets, which may limit their generalisability across different imaging systems, acquisition protocols and patient populations [46,48]. Furthermore, prospective multicentre studies assessing the impact of AI-assisted dosimetry on clinical decision-making and patient outcomes are still lacking. Although regulatory frameworks for artificial intelligence in healthcare are evolving, the pathways for the routine clinical implementation of AI-based dosimetry tools remain insufficiently defined [48,49].

From an economic perspective, the implementation of AI systems often requires significant initial investment and their cost-effectiveness depends on the size and capacity of the medical facility. As shown by El Arab et al., AI can improve diagnostic accuracy, reduce unnecessary procedures, extend QALYs and optimise resource utilisation [50]. In oncology, it has the potential to reduce both direct treatment and follow-up costs, as well as indirect costs, by minimising adverse effects and recurrence through improved treatment planning. However, as emphasised by Contaldo et al., AI complements rather than replaces healthcare professionals, which limits savings on personnel costs. Instead, it may increase efficiency and patient throughput [51,52]. Trust in AI technologies—among both clinicians and patients—is essential for successful implementation [53,54].

While simplified dosimetry and AI-based approaches show significant potential, their clinical adoption remains limited by the need for validation, standardisation and integration into existing workflows. Nevertheless, these methods may represent a key step toward the routine implementation of personalised dosimetry in PRRT.

## 3. Conclusions

The role and availability of radioisotope therapy in patients with neuroendocrine tumors are steadily increasing. Currently, most nuclear medicine departments administer fixed activities of ^177^Lu; however, these should be individualised according to patient-specific needs. Personalised dosimetry has the potential to minimise side effects and maximise therapeutic efficacy by ensuring optimal radiation delivery to target tissues while sparing healthy organs.

Although the measurement of absorbed doses is a time-consuming procedure requiring the involvement of both patients and medical staff, its role remains irreplaceable. In contrast to external beam radiotherapy dosimetry, internal dosimetry requires quantitative analysis of the patient-specific distribution of the radiopharmaceutical over time and space, taking into account physical decay and biological processes, which introduces greater measurement uncertainty.

Ongoing clinical trials and technological advancements aim to simplify and standardise dosimetric protocols. In this context, emerging tools such as artificial intelligence, deep learning and automated organ segmentation offer promising opportunities to streamline dosimetry workflows and make personalised treatment planning more accessible in routine clinical practice. However, the routine implementation of these approaches requires prospective clinical validation, standardisation of imaging and dosimetry protocols and the establishment of widely accepted dose thresholds for treatment adaptation. The development of multicentre registries and harmonised dosimetry frameworks will be crucial for generating robust evidence to support the transition from empiric fixed-activity regimens to truly personalised, dosimetry-guided PRRT [55,56].

Dosimetry enables assessment of treatment-related toxicity and represents a key element in the pursuit of personalised radioisotope therapy in the future. Increasing evidence demonstrating a clinically meaningful dose-response relationship in ^177^Lu-PRRT supports a strong scientific rationale for this transition and highlights the need for the integration of dosimetry into routine clinical practice.

## Figures and Tables

**Table 1:** Selected studies highlighting the growing clinical relevance of internal dosimetry in PRRT.

Study	Population/Setting	*n*	Dosimetry Method	Software	Key Findings	Clinical Implications
Mileva et al., 2024 [29]	Patients with GEP-NETs undergoing ^177^Lu-DOTATATE PRRT	37	SPECT/CT-based dosimetry at 3 time points	MIM software version 6.9 (MIM Software, Inc., Cleveland, OH, USA).	Demonstrated a dose–response relationship between absorbed dose and tumor control.	Early SSTR-TV decrease may help identify responders to PRRT after the first cycle.
Hebert et al., 2024 [30]	Patients with GEP-NETs undergoing ^177^Lu-DOTATATE PRRT	35	SPECT/CT-based dosimetry for cycke 1 and 2–4 time points; for cycle 3 and 4 singel time point	PLANET Dose version 3.1.1 (DOSIsoft SA, Cachan, France)	Hematologic parameters correlated with spleen and bone marrow dosimetry, whereas kidney dose did not predict renal toxicity.	Tumor and organ dosimetry may predict survival and toxicity, supporting personalised clinical management in PRRT.
Warfvinge et al., 2024 [31]	NETs treated ^177^Lu-DOTATATE	32	Hybrid planar-SPECT/CT 1d after administration SPECT and planar 1, 24, 96 and168 h	in-house software	Clear dose–response relationship in G2 NETs; ≥135 Gy predicted ~90% likelihood of partial tumor response.	Dose–response insights may inform treatment planning and optimisation of PRRT protocols.
Maccauro et al., 2024 [32]	Patients with GEP-NETs undergoing ^177^Lu-DOTATATE PRRT	42	SPECT/CT-based dosimetry at 2 time points	Dose calculations: OLINDA/EXM v1.1 (Vanderbilt University, Nashville, TN, USA)	Tumor absorbed dose correlated with progression-free survival, with a defined threshold after the first cycle identifying patients with significantly different outcomes.	Early tumor dosimetry may inform clinical decision-making and enable adaptation of treatment strategy in PRRT.
Akhavanallaf A et al., 2025 [33]	NETs treated ^177^Lu-DOTATATE at 5 centres	180	SSTR PET/CT before therapy and post–cycle 1 ^177^Lu dosimetry were conducted using centre-specific imaging protocols at 2/3 time points	Centre-specific (including MIM Software, Inc., Cleveland, OH, USA and in-house Monte Carlo software)	Renal uptake on SSTR PET correlated with absorbed dose.	SSTR PET may enable approximate prediction of kidney absorbed dose, but standardisation is essential to ensure reliable clinical use.

## Data Availability

No new data were created or analyzed in this study. Data sharing is not applicable to this article.
